# Duodenal neuroendocrine neoplasms on enhanced CT: establishing a diagnostic model with duodenal gastrointestinal stromal tumors in the non-ampullary area and analyzing the value of predicting prognosis

**DOI:** 10.1007/s00432-023-05295-9

**Published:** 2023-08-27

**Authors:** Na Feng, Hai-Yan Chen, Yuan-Fei Lu, Yao Pan, Jie-Ni Yu, Xin-Bin Wang, Xue-Ying Deng, Ri-Sheng Yu

**Affiliations:** 1 Department of Radiology, The Second Affiliated Hospital, Zhejiang University School of Medicine, Hangzhou, China; 2 Department of Radiology, Zhejiang Cancer Hospital, Institute of Basic Medicine and Cancer (IBMC), Chinese Academy of Sciences, Hangzhou, 310022 Zhejiang China; 3 Department of Radiology, The First People’s Hospital of Xiaoshan District, 199 Shixinnan Road, Hangzhou, China

**Keywords:** Duodenum, Neuroendocrine tumors, Gastrointestinal stromal tumors, Computed tomography, Survival analysis

## Abstract

**Objective:**

To identify CT features and establish a diagnostic model for distinguishing non-ampullary duodenal neuroendocrine neoplasms (dNENs) from non-ampullary duodenal gastrointestinal stromal tumors (dGISTs) and to analyze overall survival outcomes of all dNENs patients.

**Materials and methods:**

This retrospective study included 98 patients with pathologically confirmed dNENs (*n* = 44) and dGISTs (*n* = 54). Clinical data and CT characteristics were collected. Univariate analyses and binary logistic regression analyses were performed to identify independent factors and establish a diagnostic model between non-ampullary dNENs (*n* = 22) and dGISTs (*n* = 54). The ROC curve was created to determine diagnostic ability. Cox proportional hazards models were created and Kaplan–Meier survival analyses were performed for survival analysis of dNENs (*n* = 44).

**Results:**

Three CT features were identified as independent predictors of non-ampullary dNENs, including intraluminal growth pattern (OR 0.450; 95% CI 0.206–0.983), absence of intratumoral vessels (OR 0.207; 95% CI 0.053–0.807) and unenhanced lesion > 40.76 HU (OR 5.720; 95% CI 1.575–20.774). The AUC was 0.866 (95% CI 0.765–0.968), with a sensitivity of 90.91% (95% CI 70.8–98.9%), specificity of 77.78% (95% CI 64.4–88.0%), and total accuracy rate of 81.58%. Lymph node metastases (HR: 21.60), obstructive biliary and/or pancreatic duct dilation (HR: 5.82) and portal lesion enhancement ≤ 99.79 HU (HR: 3.02) were independent prognostic factors related to poor outcomes.

**Conclusion:**

We established a diagnostic model to differentiate non-ampullary dNENs from dGISTs. Besides, we found that imaging features on enhanced CT can predict OS of patients with dNENs.

## Introduction

Duodenal neuroendocrine neoplasms (dNENs) are rare heterogeneous tumors, representing about 2% of gastroenteropancreatic neuroendocrine neoplasms (GEP-NENs) and 1–3% of all duodenal tumors  (Lawrence et al. 2011; Fitzgerald et al. 2015). dNENs are divided into well-differentiated neuroendocrine tumors (NETs), including grade 1–3 (G1–3) and poorly differentiated NECs based on mitotic rate and Ki‐67 index according to the 2019 WHO classification  (Nagtegaal et al. 2020). dNENs have complex tumor biological behaviors and can range from indolent to highly aggressive in nature. According to European Neuroendocrine Tumor Society (ENETS), metastases in regional lymph nodes occur in 40–60% of dNENs at initial diagnosis  (Delle Fave et al. 2016). Prognosis of dNENs were controversially discussed due to rarity  (Delle Fave et al. 2012; Vanoli et al. 2017; Massironi et al. 2018; Folkestad et al. 2021; Nießen et al. 2020). Size, grade, depth of invasion, angioinvasion, and other factors could be related to long-term outcomes of dNENs  (Delle Fave et al. 2012; Nießen et al. 2020).

Computed tomography (CT) remains the first line imaging modality for the detection and characterization of duodenal mass-forming lesions for additional information regarding local spread and distant metastasis  (Barat et al. 2017; Jayaraman et al. 2001). dNENs are classified as ampullary and non-ampullary in location  (Delle Fave et al. 2012). Poorly differentiated NECs occur primarily in or close to the ampullary region and lead to poorer overall survival (OS)  (Vanoli et al. 2017, 2022). Signs of malignancy such as lymph node enlargement and liver metastases could be observed in imaging for ampullary NECs regardless of tumor size  (Sahani et al. 2013; Tsai et al. 2015). Instead, well-differentiated NETs are more likely to be distributed in the non-ampullary area and can mimic duodenal gastrointestinal stromal tumors (dGISTs) in enhanced CT as they often present as similar hypervascular masses  (Terra et al. 2021). dGISTs are a rare subset of gastrointestinal stromal tumors with around 3–5%  (Sugase et al. 2016) that, unlike dNENs, do not typically infiltrate adjacent structures, lack submucosal spread, and rarely metastasize to lymph nodes  (El-Gendi et al. 2012; Lee et al. 2017).

The value of contrast-enhanced CT in the diagnosis and prognosis of pancreatic neuroendocrine tumors has been widely explored (Ren et al. 2019a; Chen et al. 2022; Yang et al. 2020). A few articles have studied the imaging features of dNENs (Tsai et al. 2015; Levy et al. 2005), or their differential diagnosis with dGISTs in the ampullary region  (Ren et al. 2019b; Jang et al. 2015; Domenech-Ximenos et al. 2020). Few studies were found to focus on distinguishing them in the non-ampullary area and the value of CT in the prognosis of patients with dNENs. In this study, we aimed to establish a diagnostic model using enhanced CT to compare non-ampullary dNENs with dGISTs as well as determine the survival outcomes associated with dNENs.

## Materials and methods

This retrospective study was reviewed and approved by our institutional review board, and the requirement for informed consent was waived.

### Study population

**Fig. 1:**
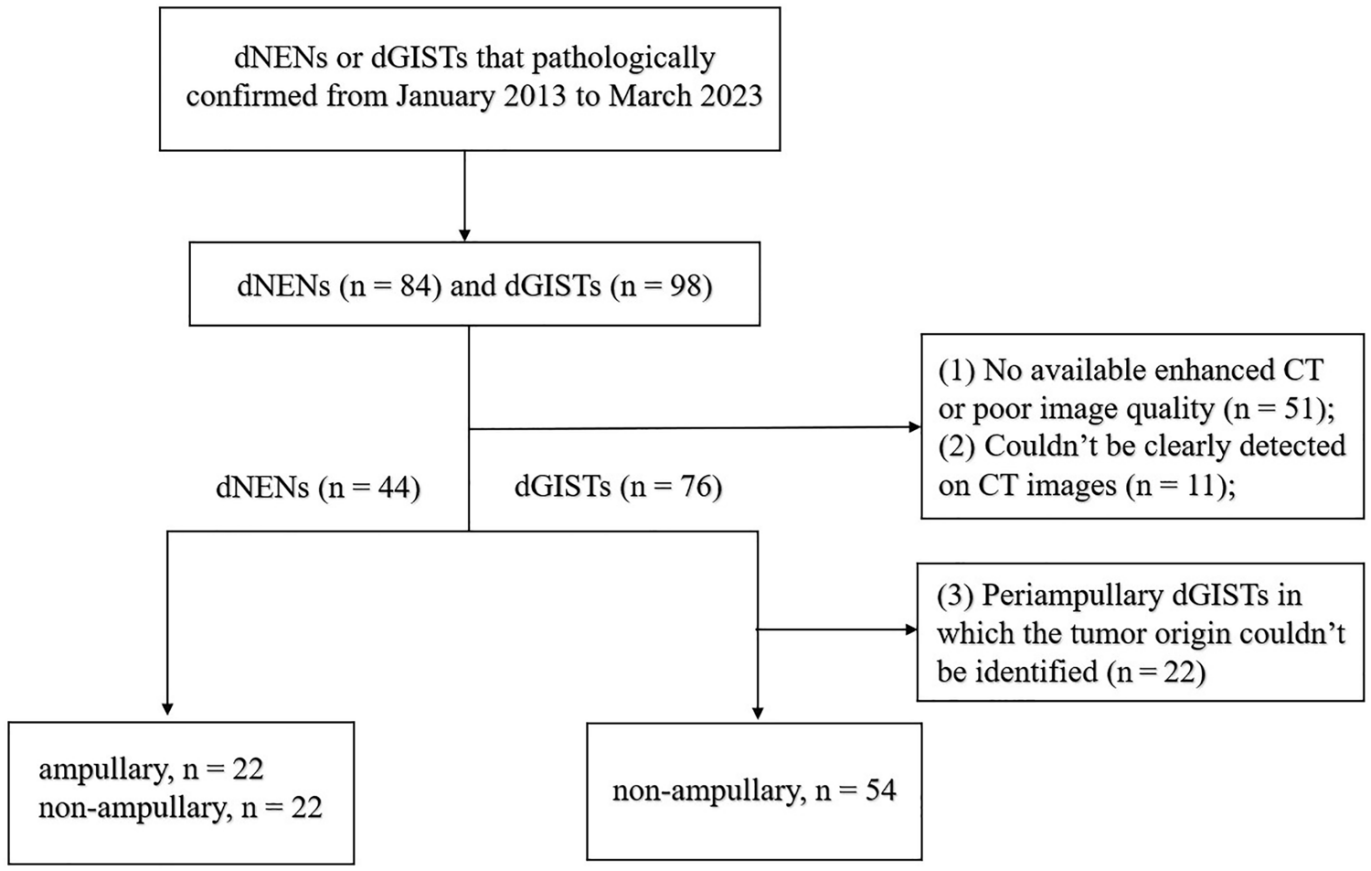
Flowchart of patient selection

Patients with pathologically confirmed dNENs (*n* = 44) and dGISTs (*n* = 54) that obtained from two independent hospitals from January 2013 up to March 2023, were retrospectively analyzed. Three patients had multiple dNENs, and the largest lesion was selected for further evaluation. The criteria for inclusion were as follows (Fig. 1): (a) patients with dNENs or dGISTs were confirmed by histopathological diagnosis obtained at biopsy or surgery; (b) patients with completed clinical data and preoperative enhanced CT images; and (c) patients who did not receive any local or systemic treatment before CT scans. The exclusion criteria were as follows: (a) no available enhanced CT or poor image quality (*n* = 51); (b) lesions could not be detected on CT images (*n* = 11) and (c) periampullary dGISTs in which the tumor origin could not be identified (*n* = 22). As a result, 45 dNENs were excluded, including 24 G1, 7 G2, 7 G3/NECs and 7 mixed carcinomas. A total of 98 patients were included, of whom 85 underwent surgical resection and 13 underwent biopsy. The specific modalities of surgery or biopsy for all patients were summarized in Tables 1 and 3.

**Table 1:** Clinical information and CT features of dNENs and dGISTs in non-ampullary area

Characteristics	dNENs (n = 22)	dGISTs (n = 54)	P value*
Age (year)	58.73 ± 9.886	57.56 ± 11.58	0.841
Gender			***0.017***
Male	16 (72.7)	23 (42.6)	
Female	6 (27.3)	31 (57.4)	
Cardinal symptoms			***0.017***
Abdominal discomfort, pain, or bloating	11 (50.0)	12 (22.2)	
Gastrointestinal bleeding (hematochezia, pale complexion, anemia)	3 (13.6)	24 (44.4)	
Other symptoms or asymptomatic	8 (36.4)	18 (33.3)	
Surgery modality			NA
Pancreatoduodenectomy/whipple surgery	7 (31.8)	7 (13.0)	
Duodenal mass resection with subtotal gastrectomy	2 (9.1)	9 (16.7)	
Limited resection^a^	7 (31.8)	33 (61.1)	
Palliative surgery	1 (4.5)	0 (0)	
Endoscopic resection	3 (13.6)	1 (1.9)	
Biopsy modality			NA
(Ultrasound-guided) endoscopic biopsy	2 (9.1)	3 (5.6)	
Laparoscopic duodenal mass biopsy	0 (0)	1 (1.9)	
Largest tumor diameter (cm)	35.72 ± 23.47	37.99 ± 19.77	0.419
Location			0.257
Bulb	9 (40.9)	14 (25.9)	
Descending	7 (31.8)	13 (24.1)	
Horizontal	5 (22.7)	17 (31.5)	
Ascending	1 (4.5)	10 (18.5)	
Growth pattern			** < *0.001***
Intraluminal	13 (59.1)	8 (14.8)	
Extraluminal	5 (22.7)	12 (22.2)	
Mixed	4 (18.2)	34 (63.0)	
Contour			0.639
Round/ovoid	9 (40.9)	19 (35.2)	
Irregular/lobulated	13 (59.1)	35 (64.8)	
Morphology			0.098
Mass	18 (81.8)	52 (96.3)	
Wall thickening with/without mass	4 (18.2)	2 (3.7)	
Ulceration			0.242
Presence	2 (9.1)	13 (24.1)	
Absence	20 (90.9)	41 (75.9)	
Tumor texture			0.263
Solid	14 (63.6)	41 (75.9)	
Solid and cystic	6 (27.3)	12 (22.2)	
Complex cystic	2 (9.1)	1 (1.9)	
Rim enhancement			> 0.999
Presence	3 (13.6)	8 (14.8)	
Absence	19 (86.4)	46 (85.2)	
Hemorrhage			> 0.999
Presence	0 (0)	2 (3.7)	
Absence	22 (100)	52 (96.3)	
Calcification			0.501
Presence	1 (4.5)	7 (13.0)	
Absence	21 (95.5)	47 (87.0)	
Border			0.138
Well-defined	17 (77.3)	50 (92.6)	
Ill-defined	5 (22.7)	4 (7.4)	
Lymph node metastases			0.128
Presence	3 (13.6)	1 (1.9)	
Absence	19 (86.4)	54 (98.1)	
Liver metastases			0.199
Presence	2 (9.1)	1 (1.9)	
Absence	20 (90.9)	53 (98.1)	
Feeding arteries			0.272
Presence	10 (45.5)	32 (59.3)	
Absence	12 (54.5)	22 (40.7)	
Intratumoral vessels			** < *0.001***
Presence	8 (36.4)	45 (83.3)	
Absence	14 (63.6)	9 (16.7)	
Draining veins			** < *0.001***
Presence	9 (40.9)	44 (81.5)	
Absence	13 (59.1)	10 (18.5)	
CT value of unenhanced lesion (HU)	41.09 ± 2.83	39.46 ± 1.90	***0.002***
Arterial lesion enhancement (HU)	116.57 ± 32.52	114.49 ± 28.47	0.909
Portal lesion enhancement (HU)	114.05 ± 23.14	110.16 ± 19.68	0.506
Enhancement in the portal phase			0.265
Isoenhancement	12 (54.5)	23 (42.6)	
Washout	5 (22.7)	13 (24.1)	
Sustained enhancement	5 (22.7)	10 (18.5)	
Mixed enhancement	0 (0)	8 (14.8)	
Arterial absolute enhancement	75.48 ± 32.08	74.84 ± 28.16	0.973
Portal absolute enhancement	72.96 ± 23.33	70.51 ± 19.24	0.740
Arterial relative enhancement ratio	1.84 ± 0.78	1.89 ± 0.71	0.663
Portal relative enhancement ratio	1.79 ± 0.60	1.78 ± 0.48	> 0.999
Enhancement grade			0.699
Mild	0 (0)	1 (1.9)	
Moderate	2 (9.1)	2 (3.7)	
Strong	20 (90.9)	51 (94.4)	
Enhancement pattern			***0.017***
Heterogeneous	11 (50.0)	42 (77.8)	
Homogeneous	11 (50.0)	12 (22.2)	

*P values < 0.05 in bold and italics indicated a statistically significant difference between groups

^a^Included wedge resection and segmental duodenectomy

G3 dNENs and NECs were not further distinguished pathologically further due to overlapping and ambiguous Ki‐67 values between them and the nature of retrospective data. As a result, G1, *n* = 12; G2, *n* = 13; G3/NECs, *n* = 19. dGISTs included the very low risk (*n* = 6), low risk (*n* = 34), intermediate risk (*n* = 3) and high risk (*n* = 11) groups according to the NIH criteria about tumor recurrence risk assessment  (Joensuu 2008).

### CT imaging acquisition

Multiple CT scanners were used as follows: TOSHIBA Aquilion 320 (TOSHIBA Medical Systems Corporation), Siemens Somatom Definition AS 6/Flash 64/Perspective (Siemens Medical Systems), Optima CT680 Series/BrightSpeed 16 (GE Medical Systems), and Ingenuity CT 64 (Philips Medical Systems). Enhanced CT images contained unenhanced, arterial, and portal venous phases for all patients. For enhanced images, an automatic power injector was used, and nonionic contrast medium (iopromide/Ultravist 370, Bayer Schering Pharma; Omnipaque 300 g/L, GE Healthcare; 100–120 mL) was administered intravenously at a rate of 3–5 mL/s. Contrast-enhanced CT images were acquired in the arterial phase at 30–40 s and in the portal venous phase at 50–70 s. CT images were obtained at 120 kVp and 150–350 mAs with a 1.5–5-mm slice thickness and a 320–380-mm field of view.

### Image analysis

Images were analyzed independently by two radiologists (Y.P. and H.Y.C., with 6 and 5 years of experience in abdominal radiology, respectively) who were blinded to patients’ pathological results. Any disagreements were resolved by consensus after consultation with a third abdominal radiologist (R.S.Y.) with over 30 years of experience.

Patients’ demographic information, including age, sex, information about cardinal symptoms, and survival outcomes were collected. OS was calculated from the date of surgery or biopsy to the date of death. The data were censored if the patient was alive at the last observed follow-up period (March 1, 2023) or if the patient was lost to follow up without reason. The qualitative CT features were collected as follows: tumor location, size (maximum diameter on axial images), morphology (mass, wall thickening with/without mass)  (Tsai et al. 2015), growth pattern, contour, ulceration, internal component (tumor texture, calcifications, hemorrhage), border, obstructive biliary and/or pancreatic duct dilation, enhancement characteristics, lymph node metastases [i.e., short-axis diameter was larger than 10 mm or included necrosis of any size  (Schwartz et al. 2016)], and liver metastases (multiple hypervascular nodules, or hypoenhancement lesions with necrosis). Enhancement characteristics included enhancement grade (difference value < 30 HU was regarded as mild, 30–50 HU as moderate, > 50 HU as strong); enhancement pattern; features of enhancement in the portal phase (isoenhancement; washout; sustained enhancement; mixed enhancement); presence of rim enhancement; feeding arteries; intratumoral vessels and draining veins. Biliary dilation was defined as extrahepatic bile duct ≥ 10 mm with/without intrahepatic duct ≥ 5 mm, and pancreatic duct dilation as main duct diameter ≥ 3 mm (Ren et al. 2020). Tumor texture was classified by the proportion of enhanced solid component in the entire tumOR as solid (solid component composed > 90%), solid and cystic (solid component composed 50–90%), or complex cystic (solid component composed < 50%)  (Ren et al. 2019b).

And then HU values of the lesion measured on triphasic CT were collected, and calculated enhancement values of tumors further: (a) arterial/portal absolute enhancement: subtract the unenhanced tumor HU value from the arterial/portal phase of the tumor HU value; (b) arterial/portal relative enhancement ratio: the arterial/portal absolute enhancement divided by the unenhanced tumor HU value. The region-of-interest (ROI) was placed carefully to avoid calcification, hemorrhage, cystic or necrotic components, vessels, and artifact areas. The quantitative data was tested two times for each lesion and then the calculated mean values were used to analyze.

### Statistical analysis

Categorical variables were presented as frequencies (percentage) and were analyzed by using Chi-square or Fisher’s exact tests. Quantitative variables were presented as mean ± standard deviations and were analyzed using a Mann–Whitney *U* test. Significant quantitative variables were dichotomized for regression analysis. Receiver operating characteristic (ROC) curve was created to determine the best cutoff values. Thereafter, binary logistic regression analyses were performed to identify the independent differential clinical or CT features. Any significant variable was retained in the final diagnostic model. Afterward, ROC curve analysis was performed to determine the diagnostic ability of the model, and the sensitivity, specificity, and 95% CI were calculated.

With regard to survival analysis, quantitative variables were dichotomized first by the best cutoff values according to ROC curves. Next, univariate Cox proportional hazard models were created to identify the risk factors for prognosis. Variables with statistical differences were then included in the forward stepwise Cox regression analysis to determine the final independent prognostic risk factors. Kaplan–Meier survival analysis with the log-rank test was used to analyze the survival outcomes among different subgroups. Statistical significance was defined with a two-sided *p* value of < 0.05. ROC curve analysis was performed using MedCalc software (version 19.8, MedCalc Software), whereas the other analyses were performed using SPSS software (ver. 25.0, IBM Inc.).

## Results

### Comparing clinical information and CT features between non-ampullary dNENs and dGISTs

The results are summarized in Table 1. There were no significant differences in age, tumor location, or tumor size between dNENs and dGISTs. In terms of gender, dNENs had a certain male predominance compared to dGISTs (72.7% [16/22] vs 42.6% [23/54]; *p* = 0.017). Moreover, patients with dGISTs were more likely to have symptoms of gastrointestinal bleeding, whereas patients with dNENs had more diverse and nonspecific symptoms.

**Fig. 2:**
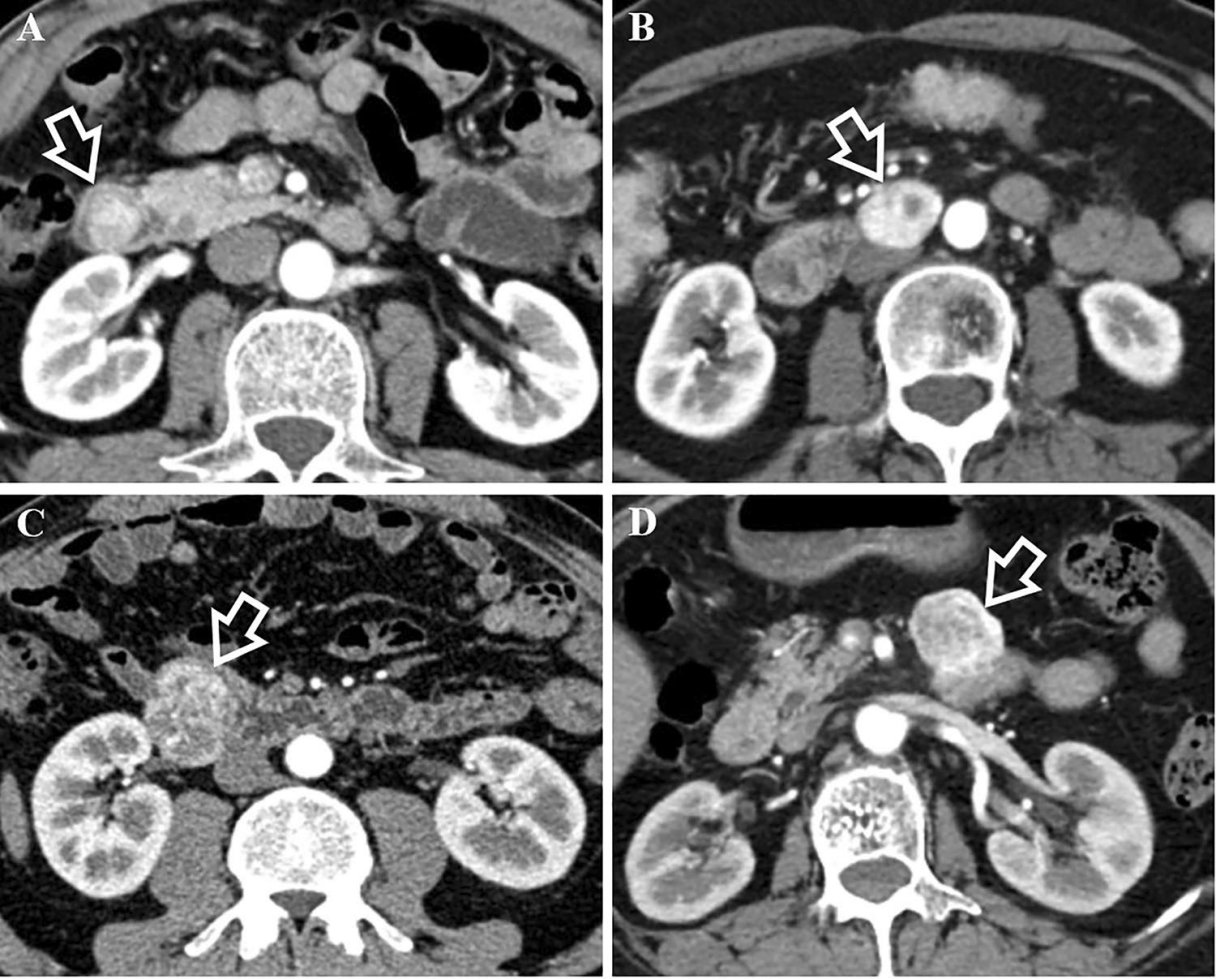
Comparison of growth pattern and intratumoral vessels in the arterial phase between dNENs (**A**, **B**) and dGISTs (**C**, **D**). **A** 57-year-old man with a G1 dNEN. CT scan shows an intraluminal nodule (white arrow) lack of intratumoral vessels in the descending part of the duodenum. **B** 59-year-old female with a G2 dNEN. There is a lesion (white arrow) lack of intratumoral vessels and presenting extraluminal growth pattern in the horizontal part of the duodenum. **C** 63-year-old male with a dGIST. It shows a lesion (white arrow) containing intratumoral vessels with mixed growth pattern in the descending part of the duodenum. **D** 66-year-old female with a dGIST. CT scan shows a lesion (white arrow) containing intratumoral vessels and presenting extraluminal growth pattern in the ascending part of the duodenum

**Fig. 3:**
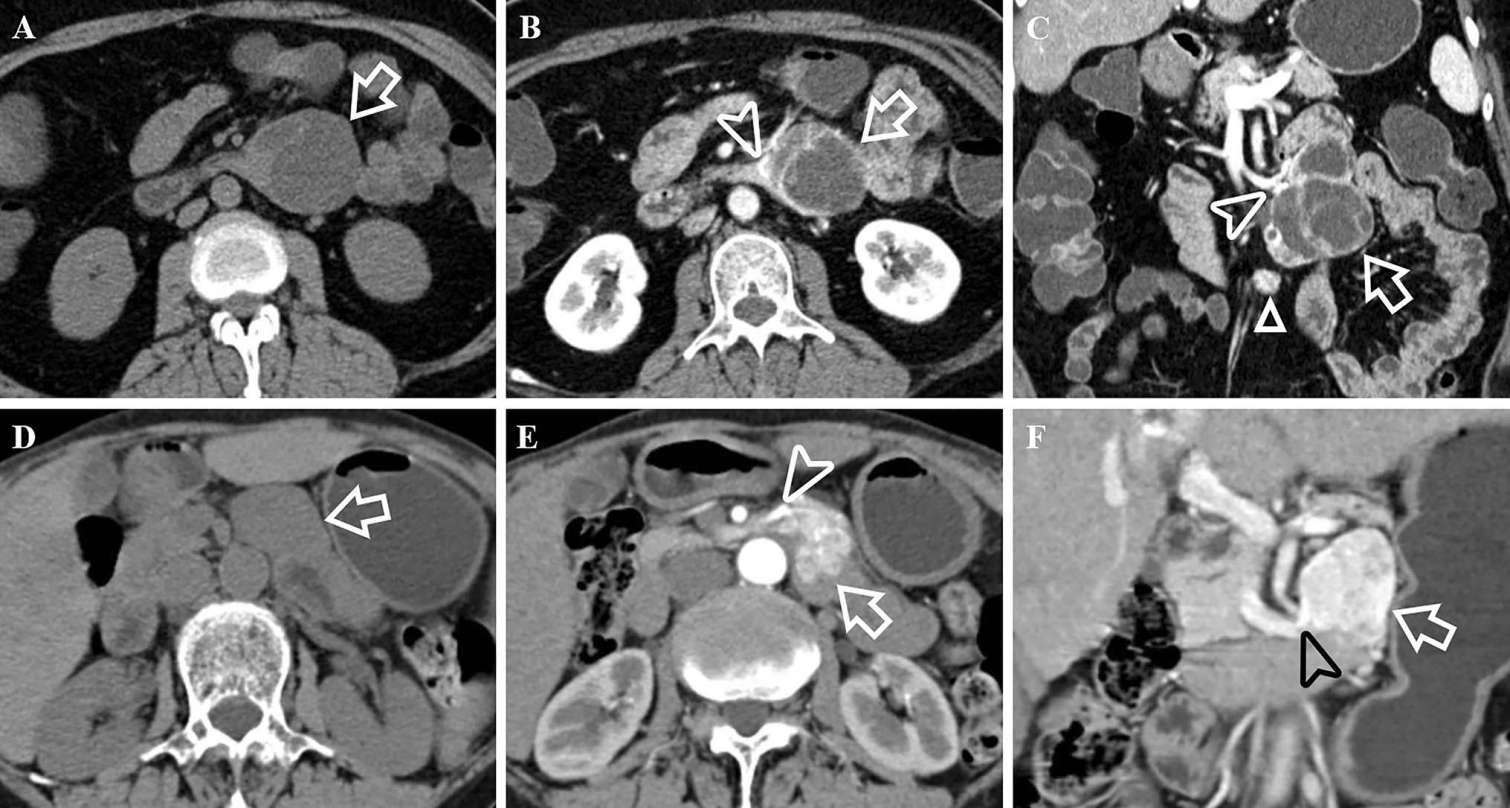
62-year-old female with a G1 dNEN. Axial image in the unenhanced phase (**A**), and axial (**B**), coronal (**C**) images in the portal venous phase show a well-defined, irregular lobulated, complex cystic lesion (white arrows) with extraluminal growth pattern in the ascending part of the duodenum. White arrowheads show that it drains to superior mesenteric vein. The feeding arteries are very slender and are therefore not shown. CT attenuation value of tumor’s solid component in the unenhanced phase is 41.85 HU. Besides, there is also a hypervascular nodule in the mesoduodenum below it (white triangle). 76-year-old female with a dGIST. Unenhanced image (**D**) and enhanced images in the arterial phase (**E**) and portal venous phase (**F**) show a well-defined, irregular, solid lesion (white arrows) with mixed growth pattern in the ascending part of the duodenum. There were abundant intratumoral vessels in the lesion. It is fed by superior mesenteric artery (white arrowhead) and drains to superior mesenteric vein (black arrowhead). CT attenuation value of the tumor in the unenhanced phase is 38.18 HU

In terms of CT features, they had significant difference in growth pattern (*p* < 0.001) with dGISTs showing prominent trend of mixed growth pattern (63% [34/54]). dNENs demonstrated intraluminal growth pattern primarily (59.1% [13/22]), of which 46.2% (6/13) showed small hypervascular intraluminal polyps less than 2 cm. With regard to tumor morphology, wall thickening was slightly more common in dNENs (18.2% [4/22] vs 3.7% [2/54]; *p* = 0.098). Containing intratumoral vessels and draining veins was not as common in dNENs as in dGISTs (for intratumoral vessels, 36.4% [8/22] vs 83.3% [45/54] [*p* < 0.001]; for draining veins, 40.9% [9/22] vs 81.5% [44/54] [*p* < 0.001]). No significant differences were found with respect to contour, ulceration, tumor texture, rim enhancement, calcification, border, lymph node or liver metastases. Regarding CT enhancement characteristics, we found that CT values of unenhanced lesions were higher for dNENs than for dGISTs (41.09 ± 2.83 vs 39.46 ± 1.90, *p* = 0.002). dGISTs were more likely to have heterogeneous enhancement than dNENs (77.8% [42/54] vs 50.0% [11/22]; *p* = 0.017). Other enhancement features revealed no significant differences. Several typical cases of dNENs and dGISTs were shown in Figs. 2 and 3.

### Establishing a diagnostic model for tumor differentiation

**Fig. 4:**
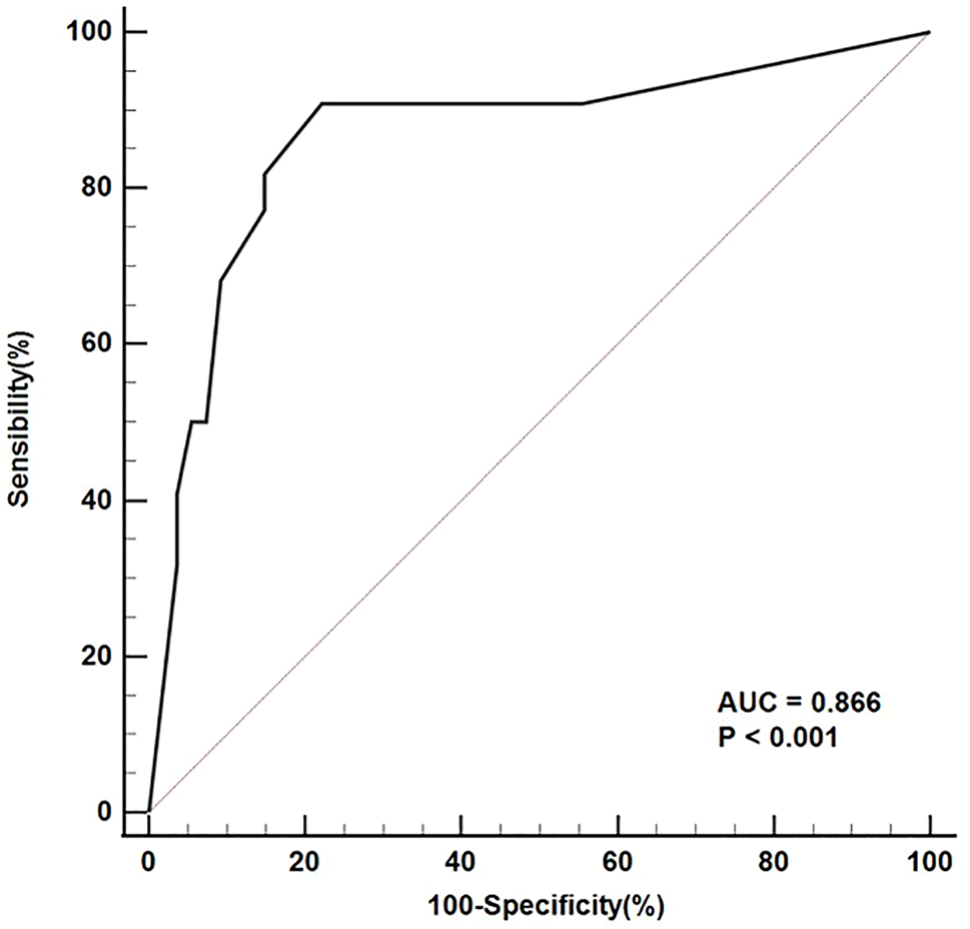
ROC curve for diagnostic model and its performance in diagnosis of non-ampullary dNENs and non-ampullary dGISTs. Diagonal line denotes reference

Among significant variables in the univariate analysis, CT value of unenhanced lesion was set to 40.76 HU as the optimal cutoff value which had 68.2% sensitivity, 79.6% specificity, 57.7% PPV, 86.0% NPV and 76.3% accuracy. Multivariate binary logistic regression analysis containing all significant variables were performed. Three variables (growth pattern, intratumoral vessels, CT value of unenhanced lesion) were considered independent predictors for differentiating dNENs from dGISTs. Intraluminal growth pattern (OR 0.450; 95% CI 0.206–0.983), absence of intratumoral vessels (OR 0.207; 95% CI 0.053–0.807) and unenhanced lesion > 40.76 HU (OR 5.720; 95% CI 1.575–20.774) were independent positive predictors of dNENs (Table 2). ROC curve analysis was performed to determine the diagnostic ability of this model (Fig. 4). The AUC was 0.866 (95% CI 0.765–0.968), with a sensitivity of 90.91% (95% CI 70.8–98.9%), specificity of 77.78% (95% CI 64.4–88.0%), and total accuracy rate of 81.58% at the optimum cut-off value. The results of Hosmer and Lemeshow chi-square testing (*χ*^2^ = 6.483; *p* = 0.262) were indicative of good calibration of the model.

**Table 2:** Multivariate regression analysis for non-ampullary dNENs diagnosis

Variables	B	P*	OR	95% CI for OR	
				Lower	Upper
Growth pattern	− 0.800	0.045	0.450	0.206	0.983
Intratumoral vessels	− 1.573	0.023	0.207	0.053	0.807
Unenhanced lesion > 40.76 HU	1.744	0.008	5.720	1.575	20.774

### Baseline data and survival outcomes of patients with dNENs

Table 3 displayed the baseline information and CT features of dNENs. A total of 44 patients were included, with 25 G1/2 dNENs and 19 G3/NECs. The overall average age was 61.11 ± 10.22 years, and 65.9% of the patients were male. The differences between G1/2 dNENs and G3/NECs were also analyzed, and all quantitative variables were dichotomized by the optimal cut-off value of ROC curves. In terms of survival outcomes, the median OS in all patients with dNENs was 61 months (range 2–90 months), 68 (range 5–73 months) for patients with G2 dNENs, and 11 months (range from 2 to 61 months) for patients with G3/NECs. The 5-year survival rate was 61.4% for the entire cohort, 100% for G1 dNENs, 84.6% for G2 dNENs, and 21.1% for G3/NECs. There were 19 deaths from any cause during the follow-up period (including 3 cases of G2 dNENs, of which 1 case died in a short time due to postoperative complications and 2 cases died of high liver tumor burden).

**Table 3:** The baseline demographic and general radiologic characteristics of all patients with dNENs and a comparison between G1/2 dNENs and G3/NEC dNENs

Characteristics	Total (n = 44)	G1/2 (n = 25)	G3/NEC (n = 19)	P value*	Cutoff value
Age (year)	61.11 ± 10.22	59.32 ± 10.10	63.47 ± 10.15	0.162	66
Gender				0.343	
Male	29 (65.9)	15 (60.0)	14 (73.7)		
Female	15 (34.1)	10 (40.0)	5 (26.3)		
Median survival time (m)	61	68	11	** < *0.001***	
Cardinal symptoms				** < *0.001***	
Diarrhea or emesis	4 (9.1)	4 16.0)	0 (0)		
Abdominal discomfort, pain, or bloating	17 (38.6)	9 (36.0)	8 (42.1)		
Obstructive jaundice (yellow urine, icterus, clay stool)	7(15.9)	0 (0)	7 (36.8)		
Gastrointestinal bleeding (hematochezia, pale complexion, anemia)	4 (9.1)	416.0)	0 (0)		
Asymptomatic	12 (27.3)	8 (32.0)	4 21.1)		
Surgery modality				NA	
Pancreatoduodenectomy/whipple surgery	21 (47.7)	11 (44.0)	10 (52.6)		
Duodenal mass resection with subtotal gastrectomy	2 (4.5)	2 (8.0)	0 (0)		
Limited resection^a^	7 (15.9)	7 (28.0)	0 (0)		
Palliative surgery	3 (6.8)	0 (0)	2 (5.3)		
Endoscopic resection	2 (4.5)	3 (12.0)	0 (0)		
Biopsy modality				NA	
(Ultrasound-guided) endoscopic biopsy	8 (18.2)	2 (8.0)	6 (31.6)		
Supraclavicular lymphadenopathy biopsy	1 (2.3)	0 (0)	1 (5.3)		
Largest tumor diameter (cm)	31.62 ± 19.67	27.32 ± 13.58	37.27 ± 24.89	0.139	26.6
Location 1				0.209	
Bulb	9 (20.5)	7 (28.0)	2 (10.5)		
Descending	25 (56.8)	11 (44.0)	14 (73.7)		
Horizontal	9 (20.5)	6 (24.0)	3 (15.8)		
Ascending	1 (2.3)	1 (4.0)	0 (0)		
Location 2				***0.001***	
Peri-/ampullary	22 (50.0)	7 (28.0)	15 (78.9)		
Non-ampullary	22 (50.0)	18 (72.0)	4 (21.1)		
Growth pattern				***0.031***	
Intraluminal	31 (70.5)	18 (72)	13 (68.4)		
Extraluminal	5 (11.4)	5 (20.0)	0 (0)		
Mixed	8 (18.2)	2 (8.0)	6 (31.6)		
Contour				0.066	
Round/ovoid	16 (36.4)	12 (48.0)	4 (21.1)		
Irregular/lobulated	28 (63.6)	13 (52.0)	15 (78.9)		
Morphology				0.219	
Mass	37 (84.1)	23 (92.0)	14 (73.7)		
Wall thickening with/without mass	7 (15.9)	2 (8.0)	5 (26.3)		
Ulceration				0.107	
Presence	8 (18.2)	2 (8.0)	6 (31.6)		
Absence	36 (81.8)	23 (92.0)	13 (68.4)		
Tumor texture				0.403	
Solid	32 (72.7)	19 (76.0)	13 (68.4)		
Solid and cystic	10 (22.7)	4 (16.0)	6 (31.6)		
Complex cystic	2 (4.5)	2 (8.0)	0 (0)		
Rim enhancement				0.337	
Presence	41 (93.2)	22 (88.0)	19 (100)		
Absence	3 (6.8)	3 (12.0)	0 (0)		
Hemorrhage				> 0.999	
Presence	1 (2.3)	1 (4.0)	0 (0)		
Absence	43 (97.7)	24 (96.0)	19 (100)		
Calcification				> 0.999	
Presence	2 (4.5)	1 (4.0)	1 (5.3)		
Absence	42 (95.5)	24 (96.0)	18 (94.7)		
Border				** < *0.001***	
Well-defined	22 (50.0)	21 (84.0)	1 (5.3)		
Ill-defined	22 (50.0)	4 (16.0)	18 (94.7)		
Obstructive biliary and/or pancreatic duct dilation				** < *0.001***	
Presence	19 (43.2)	5 (20.0)	14 (73.7)		
Absence	25 (56.8)	20 (80.0)	5 (26.3)		
Cut off suddenly of the common bile dilation				** < *0.001***	
Presence	15 (34.1)	3 (12.0)	12 (63.2)		
Absence	29 (65.9)	22 (88.0)	7 (36.8)		
Lymph node metastases				** < *0.001***	
Presence	19 (43.2)	3 (12.0)	16 (84.2)		
Absence	25 (56.8)	22 (88.0)	3 (15.8)		
Liver metastases				0.219	
Presence	7 (15.9)	2 (8.0)	5 (26.3)		
Absence	37 (84.1)	23 (92.0)	14 (73.7)		
Feeding arteries				0.976	
Presence	14 (31.8)	8 (32.0)	6 (31.6)		
Absence	30 (68.2)	17 (68.0)	13 (68.4)		
Intratumoral vessels				0.143	
Presence	17 (38.6)	12 (48.0)	5 (26.3)		
Absence	27 (52.0)	13 (52.0)	14 (73.7)		
Draining veins				0.47	
Presence	14 (31.8)	11 (44.0)	3 (15.8)		
Absence	30 (68.2)	14 (56.0)	16 (84.2)		
CT value of unenhanced lesion (HU)	41.04 ± 2.56	41.35 ± 2.88	40.64 ± 2.08	0.314	40.29
Arterial lesion enhancement (HU)	100.31 ± 32.51	112.96 ± 31.48	83.67 ± 26.26	** < *0.001***	85.33
Portal lesion enhancement (HU)	107.42 ± 21.07	116.54 ± 19.89	95.42 ± 16.28	***0.001***	99.79
Enhancement in the portal phase				0.388	
Isoenhancement	18 (40.9)	10 (40.0)	8 (42.1)		
Washout	6 (13.6)	5 (20.0)	1 (5.3)		
Sustained enhancement	20 (45.5)	10 (40.0)	10 (52.6)		
Mixed enhancement	0 (0)	0 (0)	0 (0)		
Arterial absolute enhancement	59.27 ± 32.37	71.61 ± 31.47	43.03 ± 26.33	***0.001***	42.2
Portal absolute enhancement	66.38 ± 20.90	75.19 ± 20.09	54.78 ± 15.99	***0.001***	63.02
Arterial relative enhancement ratio	1.45 ± 0.81	1.74 ± 0.78	1.06 ± 0.69	***0.001***	1.06
Portal relative enhancement ratio	1.62 ± 0.52	1.83 ± 0.52	1.35 ± 0.39	***0.002***	1.61
Enhancement pattern				***0.020***	
Heterogeneous	26 (59.1)	11 (44.0)	15 (78.9)		
Homogeneous	18 (40.9)	14 (56.0)	4 (21.1)		

*P values < 0.05 in bold and italics indicated a statistically significant difference between groups

^a^Included wedge resection and segmental duodenectomy

### Univariate and multivariate Cox regression analysis of the study population

**Fig. 5:**
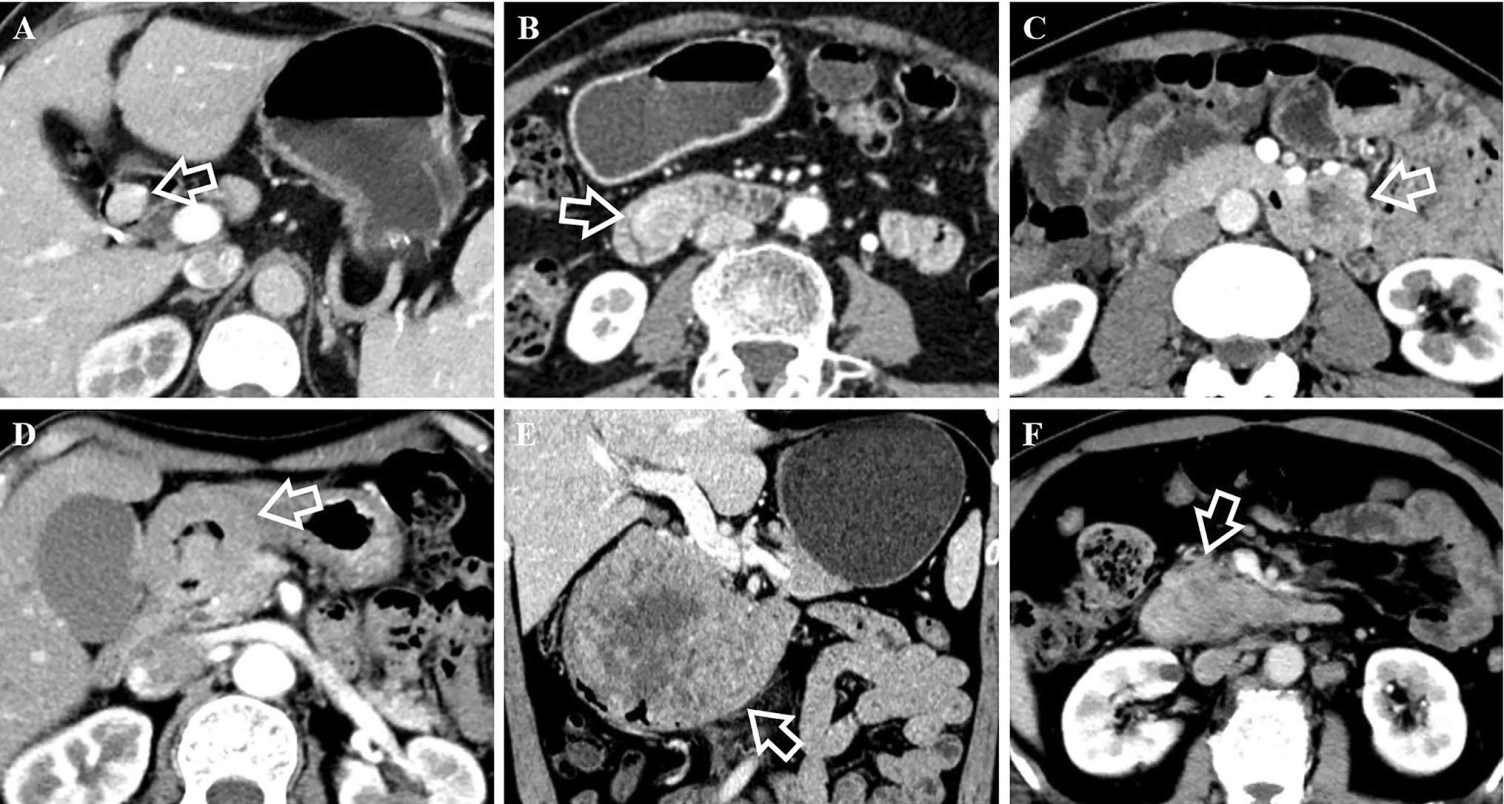
Portal venous phase enhanced CT scans of six examples of dNENs. **A** 60-year-old female with a G1 dNEN in the bulb part of the duodenum (white arrow). CT attenuation value of the tumor is 134.25 HU. Tumor recurrence was not noted during the 22-month follow-up period. **B** 74-year-old female with a G1 dNEN in the ampullary area (white arrow). CT attenuation value of the tumor is 129.16 HU. Tumor recurrence was not noted during the 10-month follow-up period. **C** 60-year-old male with a G2 dNEN in the ascending part of the duodenum (white arrow). CT attenuation value of the tumor is 128.96 HU. Tumor recurrence was not noted during the 11-month follow-up period. **D** 77-year-old male with a duodenal NEC presenting as wall thickening in the bulb part of the duodenum (white arrow). CT attenuation value of the tumor is 84.39 HU. The patient deceased at 5 months after palliative gastrojejunostomy. **E** 46-year-old male with a large G3 dNEN in the descending part of the duodenum (white arrow). The solid and cystic lesion has superficial ulceration but no obstructive biliary and/or pancreatic duct dilation. CT attenuation value of the tumor is 76.57 HU. The patient had synchronous liver metastases and was still alive during the 18-month follow-up period, but liver metastases continued to progress. **F** 65-year-old male with a duodenal NEC in the ampullary area (white arrow). The ill-defined lesion has obstructive biliary dilation. CT attenuation value of the tumor is 77.49 HU. The patient deceased at 6 months after endoscopic biopsy

**Fig. 6:**
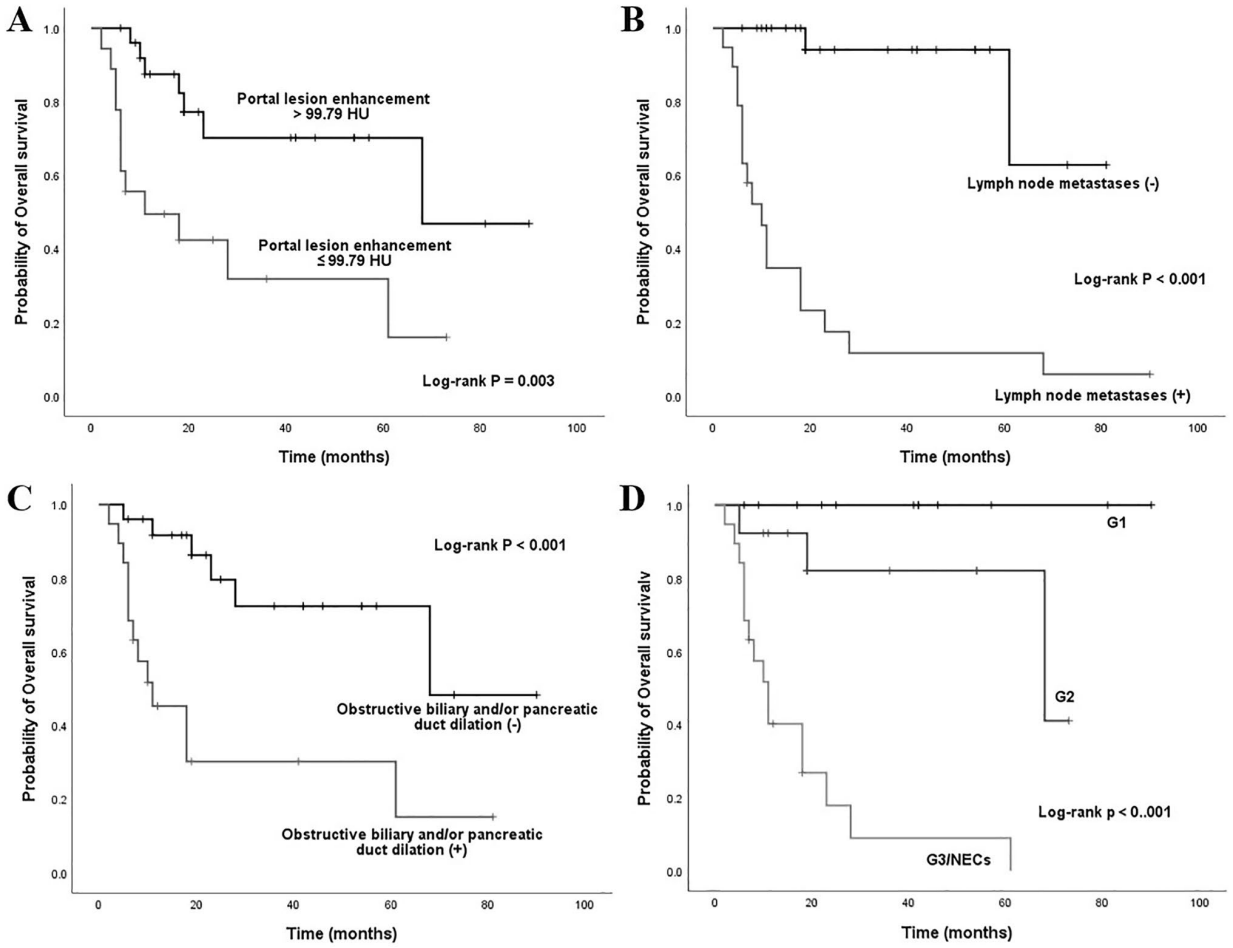
Kaplan–Meier curves for overall survival based on **a** portal lesion enhancement, **b** lymph node metastases, **c** obstructive biliary and/or pancreatic duct dilation, and **d** grade

The results of univariate Cox proportional hazard analysis were summarized in Table 4. Eighteen variables such as ill-defined border (HR: 31.321), G3/NECs (HR: 20.279), lymph node metastases (HR: 18.722), arterial absolute enhancement ≤ 42.2 HU (HR: 6.457) and so on might impact survival outcomes. These factors were then added to the multivariate Cox regression analysis. The results were detailed in Table 5 and suggest that lymph node metastases (HR: 21.602), obstructive biliary and/or pancreatic duct dilation (HR: 5.819) and portal lesion enhancement ≤ 99.79 HU (HR: 3.018) (Fig. 5**)** were independent prognostic factors related to poor outcomes. Furthermore, the Kaplan–Meier curves with log-rank of three independent prognostic factors and grade were shown in Fig. 6**.** In addition to above, we further explored the factors that related to the prognosis of ampullary dNENs. Multivariate Cox regression analysis showed that having > 3 metastatic lymph nodes (HR: 4.852, *p* = 0.016) and portal lesion enhancement ≤ 99.79 HU (HR: 5.984, *p* = 0.005) were independent prognostic factors related to poor outcomes.

**Table 4:** Univariate Cox regression analyses in patients with dNENs

Variables	Univariate analyses	
	HR (95% CI)	P value
Grade		
G1/2	1.000	
G3/NECs	20.279 (4.564–90.104)	** < *0.001***
Age (year)		
≤ 66	1.000	
> 66	0.526 (0.212–1.305)	0.166
Gender		
Female	1.000	
Male	3.716 (1.050–13.146)	***0.042***
Cardinal symptoms		
Other symptoms or asymptomatic	1.000	
Obstructive jaundice (yellow urine, icterus, clay stool)	4.339 (1.433–13.134)	***0.009***
Largest tumor diameter (cm)		
≤ 26.6	1.000	
> 26.6	3.031 (1.064–8.634)	***0.038***
Location		
Non-ampullary	1.000	
Peri-/ampullary	4.985 (1.625–15.290)	***0.005***
Contour		
Round/ovoid	1.000	
Irregular/lobulated	4.264 (1.236–14.709)	***0.022***
Morphology		
Mass	1.000	
Wall thickening with/without mass	2.465 (0.933–6.515)	0.069
Ulceration		
Absence	1.000	
Presence	2.959 (1.154–7.590)	***0.024***
Rim enhancement		
Absence	1.000	
Presence	0.043 (0.000–69.276)	0.403
Calcification		
Absence	1.000	
Presence	3.294 (0.396–27.362)	0.270
Border		
Well-defined	1.000	
Ill-defined	31.321 (4.152–236.279)	***0.001***
Obstructive biliary and/or pancreatic duct dilation		
Absence	1.000	
Presence	5.075 (1.903–13.535)	***0.001***
Cut off suddenly of the common bile dilation		
Absence	1.000	
Presence	5.692 (2.101–15.419)	***0.001***
Lymph node metastases		
Absence	1.000	
Presence	18.722 (4.281, 81.876)	** < *0.001***
Liver metastases		
Absence	1.000	
Presence	3.344 (1.245–8.980)	***0.017***
Feeding arteries		
Absence	1.000	
Presence	1.128 (0.414–3.068)	0.814
Intratumoral vessels		
Absence	1.000	
Presence	0.852 (0.321–2.261)	0.747
Draining veins		
Absence	1.000	
Presence	0.346 (0.100–1.196)	0.094
CT value of unenhanced lesion (HU)		
> 40.29	1.000	
≤ 40.29	0.994 (0.390–2.533)	0.990
Arterial lesion enhancement (HU)		
> 85.33	1.000	
≤ 85.33	6.457 (2.419–17.235)	** < *0.001***
Portal lesion enhancement (HU)		
> 99.79	1.000	
≤ 99.79	3.655 (1.430–9.338)	***0.007***
Arterial absolute enhancement		
> 42.2	1.000	
≤ 42.2	6.457 (2.419–17.235)	** < *0.001***
Portal absolute enhancement		
> 63.03	1.000	
≤ 63.02	3.368 (1.273–8.910)	***0.014***
Arterial relative enhancement ratio		
> 1.06	1.000	
≤ 1.06	6.098 (2.277–16.335)	** < *0.001***
Portal relative enhancement ratio		
> 1.61	1.000	
≤ 1.61	2.984 (0.983–9.059)	0.054
Enhancement pattern		
Homogeneous	1.000	
Heterogeneous	4.708 (1.364–16.245)	***0.014***

*P values < 0.05 in bold and italics indicated a statistically significant difference between groups

**Table 5:** Multivariate forward stepwise Cox regression analysis

Variables	HR (95% CI)	P value
Obstructive biliary and/or pancreatic duct dilation	5.819 (1.552–21.810)	0.009
Lymph node metastases	21.602 (4.193–111.299)	0.001
Portal lesion enhancement ≤ 99.79 HU	3.018 (1.007–8.454)	0.036

## Discussion

We aimed to gain insight into enhanced CT features of dNENs and established a diagnostic model containing three variables (growth pattern, intratumoral vessels, and CT value of unenhanced lesion) that can be effectively used as independent predictors to differentiate non-ampullary dNENs from non-ampullary dGISTs. Furthermore, we also analyzed positive prognostic variables that could predict the survival outcomes of patients with dNENs. Lymph node metastases, obstructive biliary and/or pancreatic duct dilation and portal lesion enhancement ≤ 99.79 HU were strong independent prognostic factors for worse outcomes in patients with dNENs. For ampullary subgroup of dNENs, having > 3 metastatic lymph nodes and portal lesion enhancement ≤ 99.79 HU were independent prognostic factors related to poor outcomes.

In this study, we focused on differentiating the two tumors in the non-ampullary region. As reported, dNENs are mostly manifested as small hypervascular intraluminal polyps and incidentally as wall thickening  (Tsai et al. 2015; Domenech-Ximenos et al. 2020; Levy and Sobin 2007), and they are predominantly located in the proximal duodenum  (Sahani et al. 2013; Levy et al. 2005). In fact, in addition to these features, 40.9% of the lesions in our study did not show intraluminal growth pattern due to being pathologically infiltrated into the subserosal/serosal layer or being located in the mesoduodenum (Figs. 2, 3, 5). As a result, these lesions usually had feeding arteries/draining veins (Fig. 3). Few studies have reported these imaging features. In contrast, dGISTs showed a prominent mixed growth pattern on CT due to arising from or between the muscularis propria and muscularis mucosa of bowel wall (Terra et al. 2021), it was consistent with the result of a previous study  (Cai et al. 2015). dGISTs have prominent feeding arteries, intratumoral vessels and draining veins  (Cai et al. 2015; Jung et al. 2020). They are usually nourished by branches of the gastroduodenal artery and(or) superior mesenteric artery supply blood, and drained into portal venous trunk and(or) superior mesenteric vein, which is primarily determined by tumor location and size. Conversely, the draining veins of dNENs were far less abundant and relatively slender, and lack of intratumoral vessels was more distinctive in this study. Moreover, unenhanced lesion > 40.76 HU was another independent predictor for dNENs diagnosis. Unlike dNENs, which originate from neuroendocrine cells in the intestinal crypt  (Kim and Hong 2016), dGISTs arise from mesenchymal tissue and usually appear as spindle cells microscopically  (Domenech-Ximenos et al. 2020; Jung et al. 2020). We speculate that the discrepancy in histological origin accounts for lower CT attenuation values in the unenhanced phase of dGISTs. In addition, no significant differences were found in CT attenuation values on the individual post-contrast phases between dNENs and dGISTs, which was consistent with a study focusing on their differentiation among small bowel neoplasms  (Shinya et al. 2017).

Some previous studies tend to differentiate dNENs from dGISTs in the ampullary area  (Jang et al. 2015), but there are significant differences in tumor biological behaviors and imaging features between them. Owing to the complex anatomy of the duodenum and pancreatic head, it may be more meaningful to distinguish periampullary dGISTs from hypovascular tumors in the pancreatic head  (Ren et al. 2019b; Jung et al. 2020). Ampullary dNENs are usually highly aggressive  (Vanoli et al. 2022), resulting in a median OS of 11 months and a 5-year survival rate of 21% in this study. Due to the involvement of the duodenal papilla, 81.8% of the ampullary lesions had biliary or pancreatic duct dilatation, which was considered as an independent predictors of poor survival outcomes in our study. Second, presence of lymph node metastases was a strong predictor of worse prognosis. Only 12% of G1/2 dNENs produced lymph node metastases due to their relative laziness, but up to 84.2% of aggressive G3/NECs did, with the latter having an extremely poor prognosis. So far, the prognostic significance of positive lymph node status has been questioned  (Folkestad et al. 2021). According to a study containing 119 cases of ampullary dNENs, having > 3 metastatic lymph nodes is a determinant of adverse prognosis in ampullary G2 dNENs  (Vanoli et al. 2022). Hence, we tested the ability of having > 3 metastatic lymph nodes to predict prognosis in the ampullary subgroup of dNENs based on this conclusion, and it turned out that this factor worked.

Some studies concluded that higher the neuroendocrine neoplasm grade result in the less intense contrast enhancement  (Tsai et al. 2015; Terra et al. 2021). Several enhancement features factors might impact survival outcomes in univariate Cox regression analyses (Table 4). Finally, only portal lesion enhancement ≤ 99.79 HU was retained as an independent prognostic factor related to poor outcomes in all dNENs patients as well as ampullary subgroup of dNENs. The value of enhanced CT in the prognosis of pancreatic neuroendocrine tumors has been widely confirmed  (Yang et al. 2020; Kim et al. 2016), which particularly highlighted the importance of portal enhancement ratio. Thus, studies with larger sample size are expected to further confirm the prognostic value of enhanced CT in dNENs.

This study has several limitations. First, we used various CT scanners and parameters due to retrospective nature. Second, we did not analyze prognosis based on more clinicopathological factors such as pathological TNM stage and angioinvasion, because we mainly focused on preoperative enhanced CT features to predict prognosis. Third, we did not measure interobserver agreement because the consensus was settled by a third radiologist. Fourth, we included several patients who underwent biopsy, which may introduce some potential bias.

In conclusion, we established a diagnostic model to differentiate non-ampullary dNENs from dGISTs. Intraluminal growth pattern, absence of intratumoral vessels and unenhanced lesion > 40.76 HU were independent positive predictors of non-ampullary dNENs. Furthermore, our study found that imaging features on triphasic CT can predict OS of patients with dNENs.

## Data Availability

The authors declare that all data and materials supporting the findings of this study are available within the article.

## Abbreviations

dNENs: Duodenal neuroendocrine neoplasms
dGISTs: Duodenal gastrointestinal stromal tumors
CT: Computed tomography
HU: Hounsfield unit
ROC: The receiver operating characteristic curve
AUC: The area under the curve
OS: Overall survival
HR: Hazard ratio
